# Ivermectin as an adjuvant to anti-epileptic treatment in persons with onchocerciasis-associated epilepsy: A randomized proof-of-concept clinical trial

**DOI:** 10.1371/journal.pntd.0007966

**Published:** 2020-01-10

**Authors:** Michel Mandro, Joseph Nelson Siewe Fodjo, Deby Mukendi, Alfred Dusabimana, Sonia Menon, Steven Haesendonckx, Richard Lokonda, Swabra Nakato, Francoise Nyisi, Germain Abhafule, Deogratias Wonya’Rossi, Jean Marie Jakwong, Patrick Suykerbuyk, Jacques Meganck, An Hotterbeekx, Robert Colebunders

**Affiliations:** 1 Provincial Ministry of Health, Bunia, Ituri, Democratic Republic of Congo; 2 Global Health Institute, University of Antwerp, Antwerp, Belgium; 3 Centre Neuro-Psycho Pathologique, University of Kinshasa, Kinshasa, Democratic Republic of Congo; 4 Center for Statistics, University of Hasselt, Hasselt, Belgium; 5 Centre de Recherche en Maladies Tropicales de l'Ituri, Rethy, Ituri, Democratic Republic of Congo; 6 Programme National de Lutte contre l’Onchocercose, Bunia, Ituri, Democratic Republic of Congo; 7 Hôpital Général de Référence de Logo, Logo, Ituri, Democratic Republic of Congo; National Institute for Infectious Diseases (L. Spallanzani), ITALY

## Abstract

**Introduction:**

Recent findings from onchocerciasis-endemic foci uphold that increasing ivermectin coverage reduces the epilepsy incidence, and anecdotal evidence suggests seizure frequency reduction in persons with onchocerciasis-associated epilepsy, when treated with ivermectin. We conducted a randomized clinical trial to assess whether ivermectin treatment decreases seizure frequency.

**Methods:**

A proof-of-concept randomized clinical trial was conducted in the Logo health zone in the Ituri province, Democratic Republic of Congo, to compare seizure frequencies in onchocerciasis-infected persons with epilepsy (PWE) randomized to one of two treatment arms: the anti-epileptic drug phenobarbital supplemented with ivermectin, versus phenobarbital alone. The primary endpoint was defined as the probability of being seizure-free at month 4. A secondary endpoint was defined as >50% reduction in seizure frequency at month 4, compared to baseline. Both endpoints were analyzed using multiple logistic regression. In longitudinal analysis, the probability of seizure freedom during the follow-up period was assessed for both treatment arms by fitting a logistic regression model using generalized estimating equations (GEE).

**Results:**

Ninety PWE enrolled between October and November 2017 were eligible for analysis. A multiple logistic regression analysis showed a borderline association between ivermectin treatment and being seizure-free at month 4 (OR: 1.652, 95% CI 0.975–2.799; p = 0.062). There was no significant difference in the probability of experiencing >50% reduction of the seizure frequency at month 4 between the two treatment arms. Also, treatment with ivermectin did not significantly increase the odds of being seizure-free during the individual follow-up visits.

**Conclusion:**

Whether ivermectin has an added value in reducing the frequency of seizures in PWE treated with AED remains to be determined. A larger study in persons with OAE on a stable AED regimen and in persons with recent epilepsy onset should be considered to further investigate the potential beneficial effect of ivermectin treatment in persons with OAE.

**Trial registration:**

Registration: www.clinicaltrials.gov; NCT03052998.

## Author summary

A proof-of-concept randomized clinical trial with a four month follow-up period, was conducted to investigate whether ivermectin had an added value in decreasing the frequency of seizures in onchocerciasis-infected persons with epilepsy who were also started on the anti-epileptic drug phenobarbital. The trial showed that ivermectin was not harmful but did not had an added beneficial effect over phenobarbital. A larger study in persons on a stable anti-epileptic treatment regimen with recent epilepsy onset should be considered to further investigate the potential beneficial effect of ivermectin treatment in persons with onchocerciasis-associated epilepsy.

## Introduction

Although onchocerciasis is classically known to only cause skin and eye disease (river blindness), epidemiological findings strongly suggest that infection with *Onchocerca volvulus* (the parasite that causes onchocerciasis) may cause a wide spectrum of seizure disorders including nodding syndrome, now described as onchocerciasis-associated epilepsy (OAE) [1]. A high prevalence of epilepsy has been reported in onchocerciasis-endemic regions, particularly in areas with ongoing onchocerciasis transmission [2]. OAE is characterized by the onset of seizures in previously healthy children between the age of 3–18 years, without an obvious cause for the epilepsy [3]. The spectrum of OAE is wide, ranging from different seizure types to Nodding and Nakalanga syndromes [3]. In a meta-analysis of eight studies in onchocerciasis-endemic African countries, Pion et al. reported that a 10% increase in the prevalence of onchocerciasis results in a 0.4% increase in epilepsy prevalence [4]. Moreover, a cohort study in Cameroon showed that children with a high density of *O*. *volvulus* microfilariae in their skin had a 28.5-fold increased risk of developing epilepsy later in life compared to children without microfilariae [5]. The pathophysiological mechanism however of OAE still needs to be elucidated. Investigations on the cerebrospinal fluid of persons with Nodding syndrome from northern Uganda suggested that autoimmune antibodies against *O*. *volvulus* could induce a neuro-inflammatory process [6]. A recent post-mortem study performed among 9 persons with OAE who died in northern Uganda showed neuro-inflammatory histopathological changes but neither *O*. *volvulus* microfilariae nor DNA were detected in the brain [7].

Accumulating evidence is suggesting a protective effect of ivermectin in the development of OAE. After the introduction of biannual mass treatment with ivermectin and ground larviciding of blackfly-infested rivers in 2012 in northern Uganda, no new nodding syndrome cases have been observed and the number of persons developing other forms of epilepsy also decreased [8]. In two age- and village-matched case control studies in the Democratic Republic of Congo (DRC), ivermectin coverage among persons with epilepsy (PWE) prior to seizure onset, was lower compared to healthy controls in the same time period [9, 10].

In a study in the Kabarole district in Uganda in 1992, 34 (37%) of 91 PWE reported some decrease in either frequency or severity of seizures after one dose of ivermectin at 150 μg/kg [11]. After being treated with ivermectin, 13 (14%) individuals had no seizures for 3.7 months (on average); seizures were unchanged in 51 (56%), and worsened in 6 (7%) [11]. It is unlikely that ivermectin will have a direct anti-epileptic effect as it does not cross the human blood brain barrier [12]. Therefore, if a reduction of seizure frequency is observed in PWE, infected with *O*. *volvulus* and treated with ivermectin, this will most likely be attributable to the ability of the latter to decrease *O*. *volvulus* microfilarial density in the PWE and/or the neurotoxic immunological response caused by the parasite.

A mathematical model predicted that microfilaridermia would be reduced by half, 24 hours after the intake of one dose of ivermectin [13]. Therefore, if microfilariae play an important role in causing OAE, it is expected that a reduction in microfilarial density following treatment with ivermectin may have a rapid influence on the seizure frequency.

To assess whether ivermectin treatment may reduce the seizure frequency and lead to seizure freedom, we conducted a four-month proof-of-concept randomized clinical trial in onchocerciasis-infected PWE. If such a trial shows a beneficial effect of ivermectin administration in PWE, this would lend further support to the hypothesis that onchocerciasis is able to cause epilepsy [14].

## Methods

### Trial design

The trial contained two treatment arms: phenobarbital + ivermectin, and phenobarbital alone. The latter will receive ivermectin treatment at the end of a 4 month follow-up period. The protocol of this study was published previously [14].

A computer-based, pre-planned age-stratified randomization list was used to randomly assign participants to one of both treatment arms in a 1:1 ratio. Subjects were assigned a randomization number by an unblinded data manager (SN) based at the University of Antwerp in Belgium, who supplied an unblinded dispenser on site in the DRC with a list of randomization numbers and corresponding treatment arms. The unblinded dispenser prepared the allocated treatment for each participant. Throughout the study period, all PWE in both arms received the anti-epileptic drug (AED) phenobarbital orally following pre-established regimens. Ivermectin was administered to the allocated study participants by the unblinded dispenser, who was not involved in assessing the participants during follow-up. All study staff involved in collecting and analyzing data were kept blinded for treatment allocation until data base lock.

### Study setting and enrolment

The trial was conducted in five onchocerciasis-endemic villages within the Logo health zone in the Ituri province, DRC: Draju, Kanga, Wala, Ulyeko and Thedeja. Mass ivermectin administration had not been implemented in the study villages. A survey by our team in 2016 documented an epilepsy prevalence of 6.2% in this area [9].

Enrolment into the trial was done during the implementation of a decentralized community-based epilepsy treatment program in the onchocerciasis-endemic zone of Logo. Before starting the recruitment of the study participants, the village chiefs, nurses and community health workers (CHW) were informed about the purpose and specificities of our study. After obtaining permission, the research team visited the study sites and set up a mobile clinic at the village health centers. Following sensitization in the target villages, persons known to have epilepsy were invited to visit the mobile clinic. All confirmed PWE went through an eligibility process before inclusion into the study. Detailed information about the trial was given in the local language Alur, and written informed consent was obtained (using thumb printing for those who could not write). All participants were enrolled in October and November 2017.

### Diagnosis of epilepsy and eligibility criteria (Table 1)

A person was considered to have epilepsy if he/she met the 2014 International League Against Epilepsy (ILAE) criteria: having experienced at least two seizures, unprovoked and without fever, with a minimal time difference of 24 hours between the two events [15].

**Table 1:** Eligibility criteria.

Inclusion criteria	Exclusion criteria
Epilepsy diagnosis confirmed by a medical doctor/neurologist	Ivermectin intake during the last 9 months
Age of 5 years and above	Pregnancy or breastfeeding
Signed informed consent form	Known or suspected allergy to ivermectin
Normal neurological development until onset of epilepsy*	*Loa Loa* microfilariae in blood
Onset of epilepsy between ages of 3 and 18 years*	Epilepsy with known cause (e.g. severe head trauma, perinatal asphyxia, history of alcohol/substance abuse, persons with a history of cerebral malaria, meningitis or encephalitis)
Seizure frequency of ≥2 seizures per month	Age less than 5 years
Presence of microfilariae in skin snip and/or Ov16 antibodies in blood	Use of AED during the two weeks prior to the trial

*Criteria suggesting onchocerciasis-associated epilepsy [2]

AED: anti-epileptic drugs

### Screening PWE for *O*. *volvulus* infection

*O*. *volvulus* infection was investigated in two ways. Firstly, the participants were tested for the presence of antibodies directed against the parasite antigen Ov16 using the Onchocerciasis IgG4 rapid test (SD BIOLINE Onchocerciasis IgG4 rapid test, Alere, Standard Diagnostics, Inc.; Yongin, Republic of Korea). Secondly, skin snips were taken from the left and right iliac crests of PWE with a Holtz corneo-scleral punch (2mm). One sterilized punch was used per subject. Each skin snip was incubated for 24 hours in isotonic saline in a well of a flat-bottom microtiter plate. The microfilaria that emerged were counted using an inverted microscope. The number of microfilariae in each well was noted recorded and the mean microfilarial density for both skin snips from the same participant was calculated and was recorded as mf/skin snip.

### Treatment regimen

All PWE were started on a phenobarbital regimen calculated as mg/body weight as follows: 5mg/kg for participants weighing <15 kg; 3mg/kg for those weighing between 15–35 kg, and 2mg/kg for participants with a weight above 35 kg. However, some PWE did not receive the exact AED dose that was prescribed because of the fluctuating availability of phenobarbital drug formulations during follow-up (30mg and/or 100mg tablets). The AED was taken orally once a day with an option to adjust the dose based on seizure frequency and/or occurrence of side effects. Individual treatment decisions were made by a neurologist (DM) and a team of physicians who had received training in epilepsy (MM, JNSF, RC, and JMJ).

In addition to phenobarbital, participants who were randomized to the phenobarbital + ivermectin arm also received one dose of 150 μg/kg ivermectin (Stromectol) at the start of the study. Ivermectin was administered orally and directly observed by the unblinded dispenser.

### Baseline and follow-up procedures

All the study procedures at baseline and during follow-up were done according to the standard operating procedures developed by the study team. At baseline, information was collected on seizure semiology, seizure frequency, epilepsy risk factors, relevant medical history, previous AED and ivermectin use. Seizures were classified according to the 2017 ILAE recommendations [16]. Weight and height measurements were carried out on all participants, followed by a complete physical and neurological examination performed by one of the medical doctors who had been trained in epilepsy, or by the neurologist. Cognitive impairment was assessed by determining whether the participant was well oriented in time and place, whether he/she could remember his/her name, was coherent in speech, and was obedient to orders; abnormal behaviour such as unexplained aggressive attitudes and/or wandering episodes were also noted.

CHW were trained to perform home visits to detect adverse effects and monitor AED adherence of the trial participants by counting pills daily for the first two weeks, and then on a weekly basis during the rest of the follow-up period. The CHW kept a seizure diary for each PWE and updated it during home visits. A research team member (DR) who had field experience in community-directed treatment with ivermectin (CDTI) supervised the community-based follow-up of participants and promptly addressed any adverse effects upon notification by the CHW. Of note, this team member was not involved in the clinical evaluation of participants during the follow-up visits.

Two weeks after randomization, a follow-up visit was scheduled to assess the early treatment outcomes and potential side effects of AED. After the second week visit, PWE had to report to the health center for further follow-up consultations by the project nurses and medical doctors, scheduled at 1, 2, 3 and 4 months. During these consultations, neurological and physical examinations were repeated, the seizure frequency assessed, adverse events evaluated, and AED usage verified. At the end of the visit, the medical doctor decided whether the initial AED treatment had to continue or needed to be adapted. CHW assisted in reminding and bringing the PWE to the health center for follow-up, and provided any relevant information collected during the home visits such as whether the PWE experienced any illness, continued to present seizures, any known seizure triggers, or any change in social status (got married, resumed work, etc.).

If a participant was unable to reach the health center, a home follow-up visit was performed by the study clinician. Phenobarbital levels in plasma were measured at month 4 to evaluate AED adherence.

### Sample size calculation

Based on previous research in Uganda (n = 476 PWE) [17], we expected that 4 months of regular AED treatment would lead to seizure freedom in 50% of the PWE. During a clinical trial performed in Rethy (Ituri province, DRC) comparing the safety and parasitological efficacy of moxidectin vs ivermectin treatment in persons with *O*. *volvulus* infection not receiving anti-epileptic treatment, 4 (80%) of 5 trial participants with epilepsy (one on ivermectin and three on moxidectin) became seizure-free for 4 months (M Mandro, personal communication).

The following null hypothesis was pre-specified: The probability to be seizure-free four months after the start of the study for immediate ivermectin treatment is equal to the probability of being seizure-free at month 4 without ivermectin treatment. If we expect that seizure freedom at month 4 will be obtained in 50% of the PWE receiving phenobarbital alone and that 80% of PWE will be seizure-free at month 4 when treated with both phenobarbital and ivermectin, about 104 subjects (52 per group) are needed to achieve a power of 90% to reject the null hypothesis at the two-sided 5% significance level. Considering that 5% of the PWE were expected to be lost to follow-up irrespective of the assigned treatment arm, 110 PWE needed to be enrolled in the trial.

### Statistical analysis

Data were analyzed based on the study arm to which the PWE were randomly allocated. SAS Software version 9.4 and R version 3.5 were used to perform the statistical analysis. Baseline characteristics were presented as count and percentage for categorical variables, while continuous variables were presented with median and interquartile range (IQR). The key primary endpoint, defined as seizure freedom at month 4, and the key secondary endpoint, defined as >50% decrease in seizure frequency at month 4, were compared between the two treatment arms at the two-sided 5% significance level.

To estimate the effect of treatment arm on the probability of seizure freedom adjusted for other factors, we constructed a multiple logistic regression model. In addition to socio-demographic features, other relevant covariates selected based on literature and/or having an influence on the outcome during univariate logistic regression analysis were included in the model. We reduced the size of the model by excluding covariates with a p-value ≥ 0.8, and consequently assessing whether their removal caused ≥20% change in the magnitude of the coefficients of the remaining covariates [18]; in which case, the excluded co-variate was considered as an important confounder and was added back into the model. Finally, we tested for all possible two-way interactions and determined their inclusion in the model by performing likelihood ratio tests. Since the goal of the trial is exploratory, rather than confirmatory, no multiple adjustments were made to control for the type I error. A similar approach was employed to evaluate the effect of ivermectin treatment on a >50% decrease of number of seizures per month compared to baseline.

Additionally, the probability of seizure freedom during the follow-up period was assessed for both treatment arms by fitting a logistic regression model using generalized estimating equations (GEE) to estimate the model parameters [19]. The model with indicators for time point (week 2, month 1, 2, 3, 4), treatment arm, and the interactions between these variables were fitted to estimate the treatment effect at each time point. A separate model with follow-up time points in months as continuous variable and the interaction with treatment arm was fitted to estimate the single parameter for the treatment effect. The best model was selected based on the quasi-likelihood information criterion (QIC) and the discrepancy between model-based and robust standard errors [20]. The model with time as continuous variable fitted better compared to one using time as a categorical variable. Fitting this model with different correlation structures, revealed that an unstructured working correlation matrix was most appropriate [19].

Given that AED doses are gradually increased in patients who do not show an improvement in their seizures, all PWE for whom the AED dose at month 4 was at least 30mg higher than the baseline dose were considered as treatment failures.

### Data handling and record keeping

All relevant clinical information was collected on paper and later entered electronically using REDCap software [21], a web-based electronic database, compliant with good clinical practice, as defined by the International Conference on Harmonization [22]. The identity and information of trial participants was kept confidential and secured in a locked cabinet at the trial site.

### Monitoring, oversight, and reporting

The trial was sponsored by the University of Antwerp. A study initiation visit was performed by the sponsor delegates before recruitment and an experienced clinical trial monitor (JM) performed the first monitoring visit on the trial site during the recruitment phase. Subsequently, because of insecurity in the region, no further on-site monitoring visits were carried out. Instead, 100% of the data were monitored internally from a distance by the data manager (SN). Moreover, 30% of the trial data, randomly selected, were double-entered using the Data Comparison Tool in REDCap. No clinically relevant discrepancies between the two entries were detected.

### Ethics

Ethical approval was obtained from the ethical committee of the School of Public Health of the University of Kinshasa in the DRC, and the ethical committee of the University of Antwerp, Belgium. The purpose and the nature of the investigation were explained to participants and parents/guardians, including risks and benefits of each of the procedures. All participants freely consented to participate after understanding the study procedures. Informed consent forms were signed/thumb printed by the subject (and parent/legal guardian if applicable), a witness, and the investigator before any procedure was performed. PWE who refused to participate were still given standard medical care and received antiepileptic treatment.

To ensure a sustainable epilepsy management plan, in collaboration with Malteser International, a decentralized program to treat all PWE in the study villages was set up. Local health personnel was trained by two neurologists and AED were made freely available.

### Trial registration

The clinical trial was registered at www.clinicaltrials.gov (NCT03052998).

## Results

### Description of the study participants

**Fig 1:**
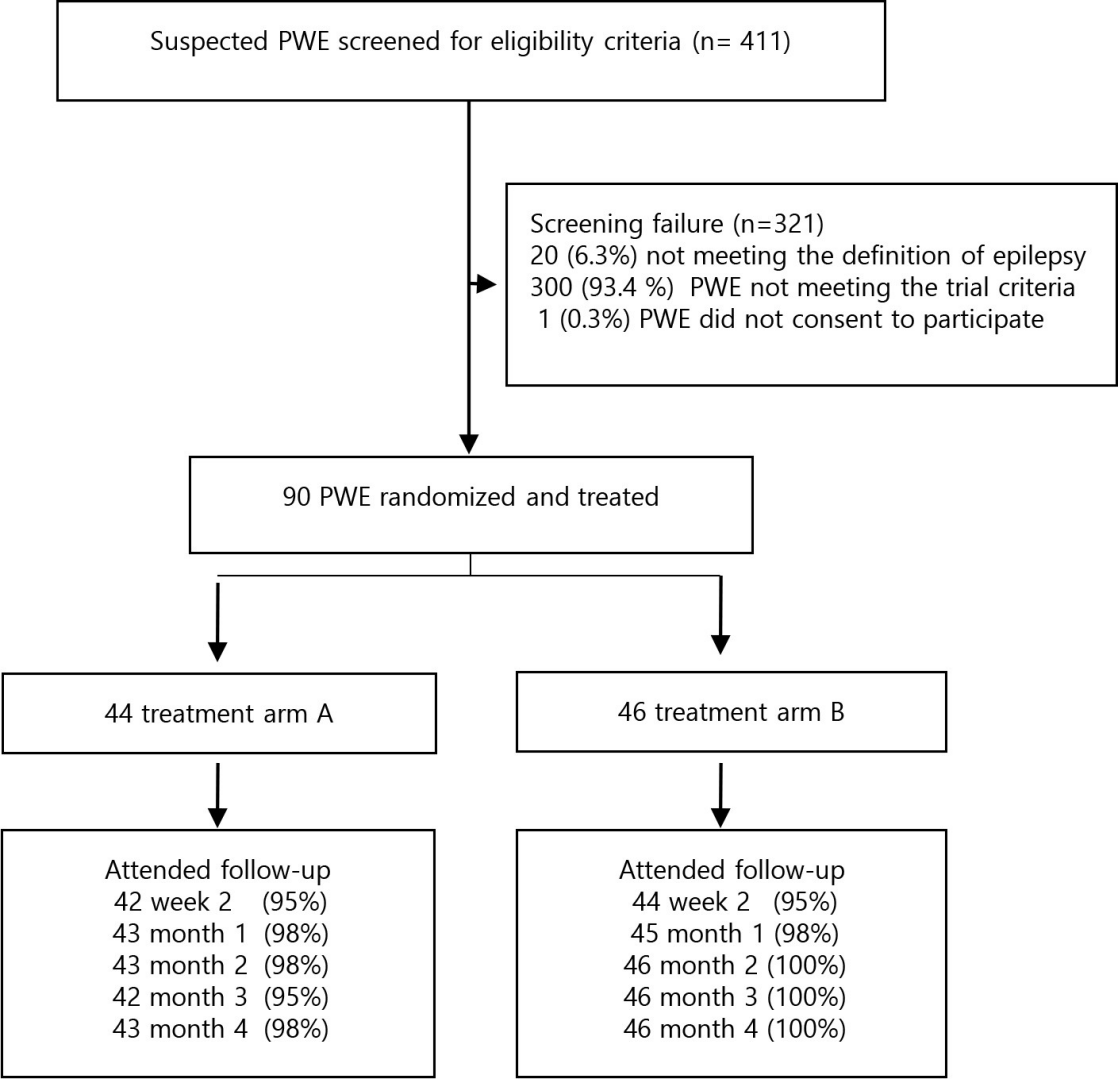
Trial participants profile.

Four hundred and eleven PWE were assessed for eligibility between October and November 2017; 321 (78.1%) were excluded for the following reasons: in 20 the epilepsy diagnosis was not confirmed, 300 did not fulfill the trial eligibility criteria, and one person refused to participate. The remaining 90 were randomly allocated to the treatment arms: phenobarbital + ivermectin (n = 44) or phenobarbital alone (n = 46). By March 2018, 89 (99%) participants in both arms attended the final follow-up visit; one participant in the phenobarbital + ivermectin treatment arm was lost to follow-up (Fig 1).

All participants belonged to the Alur ethnic group (Table 2). The majority of them were males. In the arm that only received phenobarbital, all participants presented generalized motor seizures while only 88% of PWE in the ivermectin arm had such seizures. More than 60% of participants reported to have taken AED during certain periods in the past. Although many PWE could not recall the specific AED used, phenytoin was the most frequently mentioned molecule (19/90 PWE, 21.1%). Past use of traditional treatment for epilepsy was also common. Similar proportions of participants were Ov16 and/or skin snip positive in both arms; of these, 20 (22.0%) were exclusively Ov16 positive, 25 (27.5%) were exclusively skin snip positive, and 46 (50.5%) were positive for both.

**Table 2:** Baseline characteristics of randomized participants in both treatment arms.

	Phenobarbital + ivermectin n = 44	Phenobarbital alone n = 46
Alur ethnic group, n (%)	44 (100)	46 (100)
Age in years, median (IQR)	21 (14–27)	23 (17–27)
Males, n (%) Females, n (%)	26 (59.1) 18 (40.9)	30 (65.2) 16 (34.8)
Weight in kg, median (IQR)	46 (21–55)	44 (23–56)
Generalized motor seizures, n (%)	39 (88.6)	46 (100.0)
Generalized motor seizures with absences, n (%)	19/39 (48.7)	25/46 (54.3)
Only absences, n (%)	3 (6.8)	0
Nodding seizures with and without generalized motor seizures, n (%)	4 (9.0)	3 (6.5)
Number of seizures per month, median (IQR)	3 (2–10)	4 (2–4)
Age at onset of seizures in years, median (IQR)	11 (7–14)	10 (8–13)
Duration of epilepsy, median (IQR)	9.5 (4–15)	12 (7–17)
Altered general state, n (%)	18 (41)	23 (50)
Cognitive impairment, n (%)	16 (36)	26 (56)
Behavioural abnormalities, n (%)	11 (25)	19 (41)
Use of AED in the past, n (%)	31 (70.5)	29 (63.0)
Use of traditional treatment, n (%)	17 (38.6)	22 (47.8)
Ov16 positivity, n (%)	33 (75.0)	32 (69.6)
Skin snip positivity, n (%)	32 (72.7)	35 (76.1)
Microfilarial density per skin snip, median (IQR)	15 (0.4–74.0)	18.5 (1.5–72.0)

AED: anti-epileptic drugs

IQR: interquartile range

More than 50% participants in both arms were seizure-free at month 4 (Table 3). Poor seizure control during follow-up resulted in an increased dose of phenobarbital (>30mg) for seven participants receiving phenobarbital + ivermectin, and in three receiving phenobarbital alone. The microfilarial density had significantly decreased among participants who received ivermectin. At month 4, phenobarbital serum concentration was measured in 40 participants in each arm with optimal therapeutic levels in nearly all of them (Table 3).

**Table 3:** Characteristics of participants at month 4.

	Phenobarbital + ivermectin n = 44	Phenobarbital alone n = 46
Number of seizures at month 4, median (IQR)*	0 (0–6)	0 (0–15)
Number of PWE seizure-free at month 4, n (%)	24/44 (54.5)	20/46 (43.5)
>50% decrease in seizure frequency, n (%)	30/44 (68.2)	32/46 (69.5)
Number of days without AED during the 4^th^ month, median (IQR)	0 (0–4)	0 (0–5)
Adverse event observed, n (%)	42/71 (59.2)	29/71 (40.8)
Skin snip positivity, n (%)	18/43 (41.8)	25/46 (54.3)
Microfilarial density per skin, median (IQR)**	0 (0.0–2.0)	2 (0.0–59.0)
Serum concentration of phenobarbital within therapeutic range (2–40 μg/mL) at month 4, n (%)	38/40 (95.0)	40/40 (100.0)

PWE: person with epilepsy; AED: anti-epileptic drugs; IQR: interquartile range

*Information collected for 89 participants during the last visit

**Information collected for 89 participants during the last visit; one PWE refused to provide skin snip sample.

**Fig 2:**
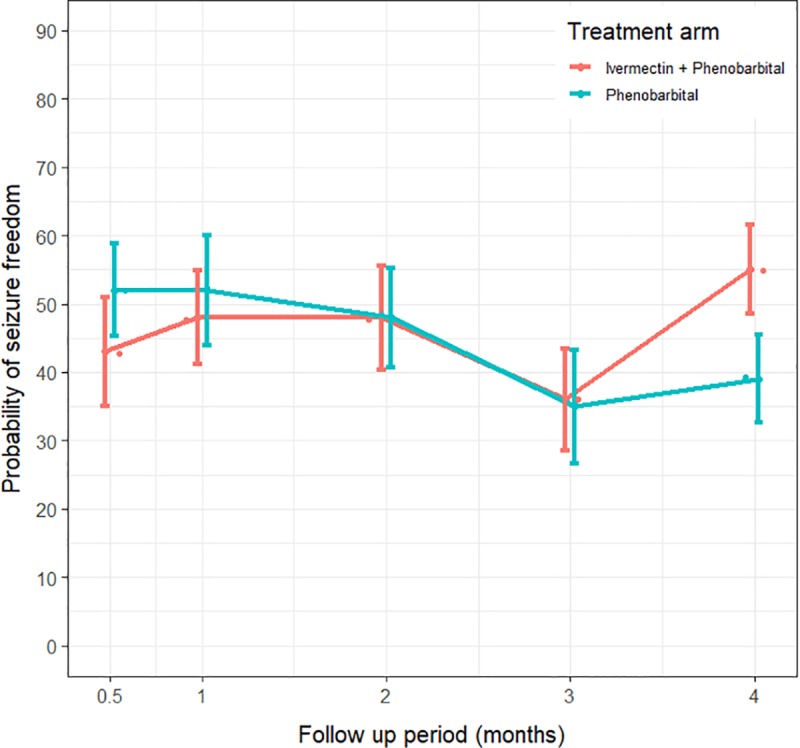
Evolution of the probability of being seizure-free in both treatment arms over time.

The probability of being seizure-free exceeded 40% in both arms after two weeks of treatment (Fig 2). At month 4, the average probability of being seizure-free for PWE treated with phenobarbital + ivermectin was 54.5%, and 43.5% among those treated with phenobarbital alone (Fig 2).

### Seizure freedom at month 4 (primary outcome)

A multiple logistic regression analysis showed a borderline association between ivermectin treatment and being seizure-free at month 4 (OR: 1.652, 95% CI: 0.975–2.799; p = 0.062) (Table 4).

**Table 4:** Multiple logistic regression exploring the association between the treatment arms and being seizure-free at month 4.

Covariates	OR	95% CI		p-value
Phenobarbital + ivermectin	1.652	0.975	2.799	0.062
Phenobarbital alone (reference)				
Female	0.768	0.452	1.306	0.331
Male (reference)				
Age	1.256	1.074	1.469	0.004
Weight (in kg)	0.961	0.903	1.023	0.210
Total number of seizures at baseline	0.969	0.893	1.052	0.458
Duration of epilepsy (years)	0.821	0.707	0.952	0.009
Dose of AED at baseline	1.134	1.017	1.265	0.024
Number of days without AED during the 4^th^ month	0.931	0.826	1.050	0.243
Microfilarial density at baseline	0.997	0.991	1.004	0.416

AED: Anti-epileptic drug; OR: Odds ratios; CI: Confidence interval

### More than 50% seizure frequency reduction from baseline value (secondary outcome)

There was no significant difference in >50% reduction of the frequency of seizures at month 4 between the two treatment arms (Table 5).

**Table 5:** Multiple logistic regression model exploring factors associated with >50% seizures reduction at month 4.

Variables	OR	95% CI		p-value
Phenobarbital + ivermectin	1.177	0.707	1.960	0.530
Phenobarbital alone (reference)				
Female	0.938	0.563	1.564	0.807
Male (reference)				
Age	1.219	1.046	1.420	0.011
Weight (in kg)	0.972	0.912	1.036	0.385
Total number of seizures at baseline	1.080	0.984	1.185	0.104
Nodding seizures	1.250	0.437	3.572	0.678
Other form of epilepsy (reference)				
Duration of epilepsy (years)	0.813	0.701	0.943	0.006
Dose of AED at baseline	1.073	1.023	1.125	0.004
Number of days without AED during the 4^th^ month	0.920	0.822	1.029	0.146
Microfilarial density at baseline	1.001	0.994	1.008	0.783

AED: Anti-epileptic drug; OR: Odds Ratio; CI: Confidence Interval

Similarly, the logistic regression model with GEE evaluating the probability of being seizure-free during the follow-up visits showed a borderline significance difference between the two treatment arms (Table 6).

**Table 6:** The parameter estimates of a GEE with unstructured correlation matrix to assess the probability of seizure freedom in the two treatment arms.

Variables	OR	95% CI		p-value
Phenobarbital + ivermectin	0.541	0.209	1.397	0.204
Phenobarbital alone (reference)				
Follow-up time (in months)	0.728	0.590	0.897	0.003
(Phenobarbital + ivermectin)*Follow-up period	1.374	1.004	1.880	0.047
Female	0.980	0.504	1.906	0.953
Male (reference)				
Age	1.091	0.984	1.210	0.097
Duration of epilepsy (in years)	0.936	0.849	1.032	0.186
Weight (in kg)	0.968	0.929	1.009	0.123
Total number of seizures at baseline	0.967	0.905	1.032	0.311
Dose of AED at baseline	1.032	1.004	1.061	0.023
Number of days without AED	0.935	0.867	1.007	0.077
Microfilarial density at baseline	1.021	0.842	1.238	0.835

*interaction term between variables

AED: anti-epileptic drugs; OR: Odds Ratio; CI: Confidence Interval

### The occurrence of adverse events in the study

A total of 71 adverse events were reported in 43 (78.9%) participants; 42/71 (59.2%) of these adverse events occurred among PWE treated with phenobarbital + ivermectin, and 29/71 (40.8%) in those treated with phenobarbital only. The most frequently reported adverse events included pruritus (16.9%), asthenia (12.7%), swelling (12.7%), somnolence (11.3%), headache (11.3%), fever (8.5%), and vertigo (8.5%) (Table 7). Two serious adverse events occurred (coma and burn), but none was related to the study drugs. Therefore, no participant was switched to a different AED.

**Table 7:** The frequency of adverse events in both treatment arms.

Adverse events	Phenobarbital + ivermectin	Phenobarbital alone	Total	% of all adverse events
Pruritus	9	3	12	16.9
Asthenia	5	5	10	14.1
Swelling	7	2	9	12.7
Somnolence	3	5	8	11.3
Headache	4	4	8	11.3
Fever	2	4	6	8.5
Vertigo	4	2	6	8.5
Left hemiplegia	0	1	1	1.4
Acute psychosis	1	1	2	2.8
Gastrointestinal disturbance	1	1	2	2.8
Arthralgia	1	0	1	1.4
Fever	1	0	1	1.4
Coma	0	1	1	1.4
Drop in libido	1	0	1	1.4
Burn	1	0	1	1.4
Injury	1	0	1	1.4
Tooth pain	1	0	1	1.4

## Discussion

This is the first randomized trial that evaluated the effect of ivermectin on the frequency of seizures in PWE with *O*. *volvulus* infection. A borderline beneficial effect of phenobarbital + ivermectin over phenobarbital alone on the probability of being seizure-free at month 4 was observed. However, there was no significant difference in the percentage of PWE with >50% reduction of seizure frequency and in the odds of being seizure-free across the follow-up visits between the two treatment arms. These inconclusive results could be due to our small sample size. Ivermectin may be able to prevent or stabilize epilepsy by lowering microfilarial density but may not be able to efficiently decrease the seizure frequency if there has been irreversible brain damage.

Our study shows that treating PWE with ivermectin was not harmful, and should therefore be encouraged. Serious acute or chronic illness were initially considered by the Mectizan donation program as contra-indications for ivermectin [23]. Currently the program does not include epilepsy as a contra-indication except during active seizure, the post-ictal period, and if they present with the Nakalanga syndrome [12]. In spite of this, we recently observed in South Sudan that certain PWE were not given ivermectin because the community considered that PWE should not take ivermectin. In villages in Maridi County in 2017, only 25.6% of PWE were treated with ivermectin [24] while the overall ivermectin coverage in persons above the age of 11 years was 50–60% [25]. Lower coverage among PWE compared to the general population has also been reported in Cameroon [26]. We equally observed that participants who presented with epilepsy of shorter duration were more likely to be seizure-free at month 4. This has already been noted previously, and highlights the need for early treatment of OAE [27].

An important observation during our study is that phenobarbital, an inexpensive drug, was able to increase the probability of seizure freedom to over 40% after only two weeks. It is worth noting that two of the three participants with absence seizures only–considered to be non-responsive to phenobarbital–were seizure-free at month four. As most persons having OAE with absence seizures and nodding seizures also develop tonic-clonic generalized seizures, phenobarbital should still be considered as a treatment option for them. Moreover, high adherence to AED treatment was achieved thanks to home visits by trained CHW. Similar results via community-based approaches have previously been reported [27]. As expected, higher AED dosages at baseline were shown to increase the likelihood to achieve seizure freedom. However, this should not justify an increase in AED dosage beyond the recommended regimens, as these may lead to side effects.

The findings of this study have to be seen in the light of a number of limitations. First, we did not include a pre-randomization period of several months of AED treatment, which would have been useful to stabilize the seizure frequency before randomization. Such a study design would have been ideal to compare the effect of ivermectin alone on the frequency of the seizures. However, we did not adopt such a design because several weeks of phenobarbital may potentially decrease the drug levels of ivermectin by affecting the P-glycoprotein (MDR1) transporter which plays a role in the elimination of the drug [28]. As per our study protocol, we initiated AED treatment at enrollment, starting with the minimal dose and increasing progressively if indicated. This is good clinical practice but complicates the data analysis because increasing the AED dose may influence the outcome parameter. Secondly, although inclusion into the study was based on fulfilling the OAE diagnostic criteria [2], some participants may have had epilepsy due to other causes than onchocerciasis, because no additional tests or imaging investigations were performed. However, since a cohort study in the Mbam Valley in Cameroon found that up to 91.7% of epilepsy cases in onchocerciasis-endemic villages were related to infection with *O*. *volvulus* [5], we do not anticipate a high frequency of PWE whose seizures are not associated with onchocerciasis among our participants. Thirdly, the fact that the study participants were not blinded as to whether they received ivermectin may have influenced the reporting of seizures by the PWE and his/her caretakers. Finally, the proposed sample size of 110 participants was not reached because of strict inclusion criteria, and this may have contributed to the lack of statistical significance in our study.

In conclusion, whether there is an added value of ivermectin in reducing the frequency of seizures in persons with OAE treated with AED remains to be determined. Ivermectin is donated for free and has little or no adverse effects on PWE. Therefore, it could be used as an additional tool to improve the quality of life and socioeconomic status of persons with OAE. A larger study in persons on a stable AED regimen and in persons with recent epilepsy onset should be considered to further investigate the potential beneficial effects of ivermectin treatment in persons with OAE.

## Supporting information

S1 File Onchocerciasis related epilepsy trial forms. (PDF)

S2 File Consort 2010checklist. (DOC)
