# Supplementary material for: Ivermectin as an adjuvant to anti-epileptic treatment in persons with onchocerciasis-associated epilepsy: A randomized proof-of-concept clinical trial
Source: PLoS Negl Trop Dis. 2020 Jan 10;14(1):e0007966. doi: 10.1371/journal.pntd.0007966 (PMC6977765; doi:10.1371/journal.pntd.0007966)
Supplement: S1 File — (PDF) [file pntd.0007966.s001.pdf]

Participant Information

Patient ID

____
(OAE suivi de 3 chiffres par exemple OAE001. OAE002)

DATE de début de l'enquête ______________________________

Nom et prenom de l'enquêteur

____
(NOM PRENOM)

ZONE de SANTE

○ Logo ○ Rethy

AIRE de SANTE

○ ulyeko ○ draju ○ kanga ○ wala ○ Tedheja

SI AIRE DE SANTE ULYEKO, QUELLE LOCALITE?

○ ulyeko ○ jupacora ○ jupadrogo ○ jupaliri ○ gulukpa ○ kor juu ○ urosi

SI AIRE DE SANTE DRAJU, QUELLE LOCALITE?

○ DRAJU ○ MAKALA ○ KPANA ○ KONDU ○ NDROY ○ RUJU ○ MBESI ○ NYODU
○ YAU ○ NZURU ○ NGBUNGBU ○ UMULO ○ JUPADROGO ○ ADRASI ○ JUPAGASA
○ JUPUKELO ○ AVE ○ PAKIDI

SI AIRE DE SANTE KANGA, QUELLE LOCALITE?

○ nguu ○ cucu ○ raa ○ kanga ○ juparima ○ wiloo ○ jabi ○ djambu ○ moo
○ THELA ○ GBANA

SI AIRE DE SANTE WALA, QUELLE LOCALITE?

○ wala centre ○ alasi ○ bugo ○ juparaja ○ juba ○ jupanjaya ○ mbafu ○ atele
○ windru ○ uguro ○ buda ○ choli ○ gomandri ○ thezii ○ bobu ○ largo
○ wiloo ○ mizaa ○ ganju ○ YUGU ○ Goma

SI AIRE DE SANTE TEDHEJA, QUELLE LOCALITE?

○ The-Deja ○ Madi ○ Ukebu Dray ○ Patole Winu ○ Jupanjaza ○ Jupudik ○ Patole
○ Juputido

NOM et PRENOM de l'INFIRMIER TITULAIRE

____
(NOM PRENOM)

NOM et PRENOM du CHEF DE GROUPEMENT

____
(NOM PRENOM)

Social Demographics

Le répondant est-il le participant lui-même ?

◯ OUI ◯ NON

Si NON, quelle est la relation entre le participant et le répondant ?

____

Un traducteur a-t-il été nécessaire ?

◯ OUI ◯ NON

Date de le naissance connue?

◯ OUI ◯ NON

Date de naissance, si connue

____
(ne doivent pas être âgés de moins de 5)

Age

____
(YEARS)

Age, si date de naissance pas connue

____
(YEARS)

Age group

◯ age < =20 ◯ age>20

Sexe

◯ mâle ◯ Femelle

Ethnie

◯ Alur ◯ Lendu ◯ Ndookebo ◯ Other

Si autre précisez

____

Poids

____
(Kg)

Taille

____
(CM)

Niveau scolaire

◯ Aucun ◯ Primaire ◯ Secondaire ◯ Superieur ◯ Ne sait pas

If Primaire

◯ 1 ◯ 2 ◯ 3 ◯ 4 ◯ 5 ◯ 6

If secondaire

◯ 1 ◯ 2 ◯ 3 ◯ 4 ◯ 5 ◯ 6

Certificat d'étude primaire

◯ Oui ◯ Non ◯ Ne sait pas ◯ NA

Est-ce que le participant a déjà pris Ivermectine/Mectizan (médicament distribué contre la cécité):

◯ oui ◯ non ◯ NE SAIT PAS

Si oui, combien de fois? _________ nombre de fois

____
(nombre de fois)

Si oui, en quelle année ?
(ne pas oublier, pour les femmes d'insister sur les années de grossesses car souvent elles oublient qu'elles ne l'ont pas pris durant leur grossesse).

	2017	2016	2015	2014	2013	2012	2011	2010
quelle annne	☐	☐	☐	☐	☐	☐	☐	☐

Quand le participant a pris la dernière fois Ivermectine/Mectizan?

____
(annee)

Quand le participant a pris la dernière fois Ivermectine/Mectizan?

____
(Mois)

Medical History

Est-ce que le participant a éprouvé ou noté un ver se déplaçant dans / le long de la partie blanche de son il

◯ oui ◯ non ◯ NE SAIT PAS

ENQUETEUR : Montrer une photographie du ver de l'

◯ oui ◯ non ◯ NE SAIT PAS

Si, oui, la dernière fois que vous avez eu ce problème, combien de jours avez-vous vu le ver avant qu'il ne disparaisse?
(NE PAS proposer de réponse au participant, attendre sa réponse)

◯ Jours ◯ Ne sait pas

Si Jours,

____
(Jours)

Accouchement (naissance du participant enquêté), mère présente pour répondre aux questions ?

◯ OUI ◯ NON

Lieu de naissance du participant enquêté ?

◯ Domicile ◯ Centre de Santé ◯ Hôpital ◯ Autre ◯ NE SAIT PAS

Accouchement

◯ normale ◯ travail difficile ◯ par césarienne ◯ ne sait pas

A LA NAISSANCE

Le participant enquêté est-il né prématuré ?

◯ oui ◯ non ◯ NE SAIT PAS

Âge du patient à l'apparition des premières crises de l'épilepsie

____
(Years)

Si depuis moins d'1 an, depuis combien de mois?

____
(mois)

Si age exact de début de l'épilepsie pas connue, estimer groupe d'age.

◯ < 3 years ◯ 4 to 18 years ◯ >18 years

Est-ce qu'avant l'épilepsie le participant, a souffert ?

	Oui	Non	Ne sait pas
traumatisme crânien?	◯	◯	◯
encéphalite/méningite?	◯	◯	◯
malaria sévère ?	◯	◯	◯
rougeole sévère	◯	◯	◯
maladie du sommeil ?	◯	◯	◯
autre maladie grave	◯	◯	◯

si autre maladie, spécifier

____

CONVULSIONS FEBRILE dans l'ENFANCE (0-10ans).

◯ oui ◯ non ◯ NE SAIT PAS

Si OUI : à quel âge ont-elles débuté ?

____

Perte de connaissance avec convulsions non provoquée par la fièvre?

◯ Jamais ◯ 1X ◯ 2X OU PLUS ◯ NE SAIT PAS

Perte(s) de connaissance avec perte(s) d'urine ?

◯ oui ◯ non ◯ Non fait ◯ Not Known ◯ NE SAIT PAS

Absence(s) ou perte(s) de contact avec l'entourage (participant ne réponds pas, de début brutal et de durée brève) ?

◯ oui ◯ non ◯ NE SAIT PAS

Perte(s) de connaissance avec émission de bave et/ou morsure de langue?

◯ oui ◯ non ◯ NE SAIT PAS

Episodes de hochement de tête durant perte de contact avec l'entourage ?
(participant ne réponds pas, et hochement non suivi de convulsions)

◯ oui ◯ non ◯ NE SAIT PAS

Secousses ou mouvements anormaux incontrôlables d'un ou des membres ?
(convulsions., de début soudain et d'une durée de quelques minutes)

○ oui ○ non ○ NE SAIT PAS

Apparition brutale et de durée brève de sensations corporelles étranges, d'hallucinations ou d'illusions visuelles, auditives ou olfactives ?

○ oui ○ non ○ NF ○ NE SAIT PAS ○ NA

A-t-on déjà dit à la personne qu'elle était épileptique ou qu'elle avait déjà fait des crises d'épilepsie ?

○ oui ○ non ○ NE SAIT PAS

Le participant a-t-il présenté une crise dans les 5 dernières années

○ oui ○ non ○ NE SAIT PAS

Le participant a-t-il présenté une crise la dernière année ?

○ oui ○ non ○ NE SAIT PAS

Le participant a-t-il présenté une crise le dernier mois ?

○ oui ○ non ○ NE SAIT PAS

TYPE D'ÉPILEPSIE

Crises généralisées motrices

○ OUI ○ NON

si, oui

○ tonico-cloniques ○ atoniques ○ autre

Si,autre, specifier

Absences

○ OUI ○ NON

Crises avec debut focales sans de perte de connaissance

○ OUI ○ NON

Si oui, Crises avec debut focales sans de perte de connaissance?

○ motrices ○ autre

Si, autre, specifier

Crises avec debut focal avec perte de connaissance

◯ OUI ◯ NON

Si oui ?

◯ motrices ◯ autre

Si autre ,specifier

Crises avec debut focal secondairement bilateralement tonico-clonique

◯ OUI ◯ NON

Hochement de tête (avec période brève de perte contact)

◯ oui ◯ non ◯ NE SAIT PAS

Quel facteur peut déclencher une crise d'epilepsie ?

	Oui	Non	Ne sait pas
pas de facteur spécifique	◯	◯	◯
la nuit	◯	◯	◯
le fain	◯	◯	◯
le froids	◯	◯	◯
durant repas	◯	◯	◯
les orages	◯	◯	◯
autre	◯	◯	◯

Si autre, spécifier

AVANT LE DÉBUT DE L'ÉPILEPSIE

Comparé aux enfants de même âge que lui, comment on apprécierait son développement?

Capacité de marcher ?

◯ tôt ◯ même âge ◯ plus tard ◯ ne sait pas

Capacité de parler avec des phrases ?

◯ tôt ◯ même âge ◯ plus tard ◯ ne sait pas

Est-ce que l'enfant a grandi

◯ plus vite ◯ normalement ◯ moins vite ◯ ne sait pas

APRÈS LE DÉBUT DE L'ÉPILEPSIE
Comparé aux enfants de même âge que lui, comment on apprécierait son développement ?

Est-ce que l'enfant a grandi

◯ plus vite ◯ normalement ◯ moins vite ◯ ne sait pas ◯ NA

Combien de crises d'épilepsie?(nombre totale d'ecrises abscence plus convulsions)

	Oui	Non	Ne sait pas
chaque jour (plus que 30 par mois)	◯	◯	◯
chaque mois (moins de 30 par mois)	◯	◯	◯
chaque année	◯	◯	◯

combien de fois par jour

____

combien de fois par mois

____

combien de fois par ans

____

ANAMNESE

Combien de crises d'épilepsie pendant le dernier mois ?

Convulsions généralisées :

____

Absences

____

Autres

____

Nombre total de crises

____

Est-ce que vous prenez des médicaments MODERNES contre l'épilepsie?

◯ OUI, de façon irrégulière ◯ OUI, après chaque crise ◯ OUI, continuellement ◯ NON, jamais

Si prise d'antiépileptiques modernes, lesquels ?

	OUI	NON	NE SAIT PAS
phénobarbital	◯	◯	◯
carbamazepine	◯	◯	◯
phenotoine	◯	◯	◯
sodium valproate	◯	◯	◯
Autre	◯	◯	◯

Quelle dose phenobarbital?

____

fois par jour

____

Quelle dose carbamazepine ?

____

nombre de fois par jour

____

Quelle dose phenotoine

____

nombre fois de jour

____

Quelle dose sodium valproate

____

nombre de fois par jour

____

si autre,spécifier

____

Est-ce que vous prenez des médicaments TRADITIONNELS contre l'épilepsie?

◯ NON, jamais ◯ OUI, de façon irrégulière ◯ OUI, continuellement

Est-ce qu'il y a d'autres personnes avec épilepsie dans la famille (vivant la même maison)

◯ oui ◯ non ◯ NE SAIT PAS

Si OUI, préciser :

◯ père ◯ mère ◯ frère ou s□ur

Préciser le nombre de frere ou soeur avec epilepsie

____

Combien de membres de la famille(au même village) souffrent d'épilepsie (avec vous enclus)?

____
(avec vous enclus)

Combien âgé de < 20 ans

____
(avec vous enclus)

Le participant enquêté a-t-il un jumeau ou une jumelle identique ?

◯ oui ◯ non ◯ NE SAIT PAS

Si oui, est-ce que le jumeau souffre d'épilepsie ?

____

Evaluation de la qualité de vie

	sans problème	petit problème	grand problème	NA
Activités ménagères/champêtres	◯	◯	◯	◯
Degré d'autonomie	◯	◯	◯	◯
jeux	◯	◯	◯	◯
socialisation/causeries	◯	◯	◯	◯
Satisfaction/joie de vivre	◯	◯	◯	◯
Anxiété/peur de nouvelle crise	◯	◯	◯	◯
Performances scolaires(Si scolarisé)	◯	◯	◯	◯

Physical Examination

EXAMEN PHYSIQUE

	Bonne Sante	Moderement altere	Mauvais
ETAT GENERAL(Nutritionel)	◯	◯	◯

Please indicate if these examinations were done?

	OUI	NON
VISION NORMALE	◯	◯
yeux vitreux	◯	◯
AVEUGLE DES YEUX	◯	◯
CICATRICES DE BRULURES	◯	◯
PEAU NORMALE	◯	◯
PAPULES PRURIGINEUX	◯	◯
PEAU DE LEOPARD	◯	◯
PEAU DE LEZARD	◯	◯
DEMANGEAISONS	◯	◯
NODULES ONCHOCERCIENS	◯	◯
CANDIDOSE BUCCALE	◯	◯
SIGNES MORSURE DE LANGUE	◯	◯
GANGLIONS CERVICAUX ANORMAUX (>1cm)	◯	◯

if nodules onchocerciens, nombre

____

Adolescent (>16 ans) ou adulte qui ressemble à un enfant

◯ oui ◯ non ◯ NA

19.3 Si OUI, spécifier

◯ Filles ◯ garçons

Si,Filles

	OUI	NON
seins développé	◯	◯
poils sur le pubis	◯	◯

	Oui	Non
THORACIQUE	◯	◯
SCOLIOSE	◯	◯
KYFOSE	◯	◯
VISAGE	◯	◯
autre	◯	◯

si,autre deformation corporelle

____

CONSCIENCE

◯ Alerte ◯ Confus ◯ réagi aux stimuli verbaux ◯ réagi aux stimuli douloureux ◯ Ne réagi pas

TROUBLE MENTALE ?:

◯ NON ◯ Peu important (dérange les activités mais encore actif) ◯ Important (empêche d'exercer les activités)

Si trouble mental (important ou peu important): lequel? (plusieurs réponses possible)

	Oui	Non	Ne sait pas
difficultés de s'exprimer	◯	◯	◯
désorientation dans le temp	◯	◯	◯
désorientation dans le space	◯	◯	◯
hallucinations	◯	◯	◯
exprime des idées délirantes	◯	◯	◯
ne comprends pas ce qu'on demande	◯	◯	◯
épisodes agressifs	◯	◯	◯
oublie facilement	◯	◯	◯
troubles de comportement	◯	◯	◯
peu d'intérêt ou plaisir dans presque toute activité	◯	◯	◯
autre	◯	◯	◯

Autre

____

Mouvements oculaires

◯ Normale ◯ altérée ◯ NA

Epreuve doigt-nez

◯ Normale ◯ altérée ◯ NA

Marche ?

○ normale ○ base élargie ○ spastique ○ hémiplégique ○ autre

si,autre specifier

____

Faiblesse musculaire ?

○ Non ○ Généralisée ○ Localisée

Si oui, spécifier

____

Tonus

○ normale ○ hypotonie ○ hypertonie

Reflexes tendineux :

○ normaux ○ faibles ○ hyper réactifs ○ NF ○ NA

Babinsky

○ présent ○ absent ○ NF ○ NA

EXAMEN NEUROLOGIQUE :

○ NORMAL ○ ABNORMAL

Diagnostique

☐ Epilepsie ☐ Syndrome du hochement de tète ☐ Hochement de tête et autre forme d'épilepsie
☐ autre

23.2. Si autre, spécifier :

○ Convulsions fébriles récurrentes ○ Syncopes ○ Vertiges ○ Anémie sévère ○ retard mental sans épilepsie ○ Hypoglycemie ○ Problème psychiatrique ○ retard mental aved épilepsie
○ nakalanga ○ abscences

PRELEVEMENTS / TESTS

SANG

○ OUI ○ NON

Cocher la/les case(s) adaptée(s) au prélèvement :
(Cocher la/les case(s) adaptée(s) au prélèvement :)

☐ tube SSTTM II ☐ tube EDTA

BIOPSIE CUTANEE EXANGUE (SKIN SNIP).

◯ OUI ◯ NON

Date de la biopsie

____
(DD-MM-YYYY)

Heure de la biopsie

____
(HH:MM)

Si OUI, combien de prélèvements ont-ils été effectués ?

☐ Droite ☐ Gauche

Date de lecture

____
(DD-MM-YYYY)

Heure de lecture

____
(HH:MM)

Microfilaire/mg de peau droite

____

Microfilaire/mg de peau gauche

____

Biopsie cutanée préservée en tube rempli d'éthanol 80% au moins

◯ OUI ◯ NON

GOUTTE EPAISSE CALIBREEpour diagnostic de la LOASE (Loa Loa)

◯ POSITIF ◯ NEGATIF ◯ NON EFFECTUE

Si POSITIF, valeur de lecture :

____

Signature MD/personnel santé

____

Date of signature

____

Selection du participant a l' etude clinique

Diagnosis Epilepsy

Epilepsie

◯ OUI ◯ NON

Inclusion criteria

Age de 5 ans ou plus

◯ OUI ◯ NON

Onset of Epilepsie between age of 3 et 18 ans

◯ OUI ◯ NON

Greater than 2 seizures per month

◯ OUI ◯ NON

Exclusion Criteria

Traitement par l'Ivermectine durant les 9 derniers mois

◯ OUI ◯ NON

Allaitement maternel < 8 jours /Enceinte

◯ OUI ◯ NON

Allergie a l'ivermectine

◯ OUI ◯ NON

Contre-indication à l'utilisation des antiépileptiques, le phénobarbital, la carbamazépine ou sodium valproate

◯ OUI ◯ NON

Infection concomitante à Loa Loa

◯ OUI ◯ NON

Epilepsie avec une cause connue
(par exemple un traumatisme crânien grave, l'asphyxie périnatale ou des antécédents de paludisme cérébral ou de méningite / d'encéphalite)

◯ OUI ◯ NON

Autre maladie aiguë concomitante ou l'utilisation chronique de médicaments

◯ OUI ◯ NON

Utilisation de medicaments antiepileptiques durant les 2 derniers semaines

◯ OUI ◯ NON

Diagnostic Onchocercose

OV 16 positif

◯ OUI ◯ NON

Biopsie cutane positive

◯ OUI ◯ NON ◯ WAITING FOR RESULTS

Onchocercose positive

◯ OUI ◯ NON

GROUP

____

Randomisation Form

Patient meets all inclusion criteria and no exclusion criteria and Consentement éclairé obtenu

◯ OUI ◯ NON

Randomisation Group 1

◯ Group 1A ◯ Group 1B

SIGNATURE

____

Date the randomisation form was signed

____
(la date doit être fournie par randomiser)

Week2 Follow Up Form

ANAMNESE

Combien de crises d'épilepsie pendant les 2 dernieres semaines ? Utilisez le journal des crises.

____

Convulsions généralisées :

____

Absences

____

autre

____

Nombre total de crises

____

Description type et severite des crises

	Oui	Non
Phénobarbital	○	○
Carbamazépine	○	○
Valproate de sodium	○	○
Aucun	○	○

Si, Phénobarbital a quelle dose?

____
(mg)

fois de jour

____

Si, Carbamazépine a quelle dose?

____
(mg)

fois de jour

Si, Valproate de sodium,a quelle dose?

fois de jour

Si,aucun, pourquoi?

Est-ce que les médicaments ont été pris selon la prescription ? Utiliser la saisie journalière / comptage des comprimés

○ OUI ○ NON

Nombre de comprimés recu................

Nombre de comprimés pris................

Si, Non, nombre de comprimés oubliés

(Utiliser la saisie journalière / comptage des comprimés)

Nombre de jours sans médicament

(jours)

Date de la signature

Follow up Form

Le répondant est-il le participant lui-même ?

◯ OUI ◯ NON

Si NON, quelle est la relation entre le participant et le répondant ?

____

Un traducteur a-t-il été nécessaire ?

◯ OUI ◯ NON

ANAMNESE

Combien de crises d'épilepsie pendant le dernier mois ? Utilisez le journal des crises.

____

Convulsions généralisées :

____

Absences

____

autre

____

Nombre total de crises

____

Description type et severite des crises

Evaluation de la qualité de vie

	Amélioration	Dégradation	Idem	NAP
Activités ménagères/champêtres	◯	◯	◯	◯
Degré d'autonomie	◯	◯	◯	◯
Jeux	◯	◯	◯	◯
Socialisation/causeries	◯	◯	◯	◯
Satisfaction/joie de vivre	◯	◯	◯	◯
Anxiété/peur de nouvelle crise	◯	◯	◯	◯
Performances scolaire (Si scolarisé)	◯	◯	◯	◯

	Oui	Non
Phénobarbital	◯	◯
Carbamazépine	◯	◯
Valproate de sodium	◯	◯
Aucun	◯	◯

Si, Phénobarbital a quelle dose?

____
(mg)

fois de jour

____

Si, Carbamazépine a quelle dose?

____
(mg)

fois de jour

____

Si, Valproate de sodium,a quelle dose?

____

fois de jour

____

Si,aucun, pourquoi?

____

Est-ce que les médicaments ont été pris selon la prescription ? Utiliser la saisie journalière / comptage des comprimés

◯ OUI ◯ NON

Nombre de comprimés recu.................

____

Nombre de comprimés pris.................

____

Si, Non, nombre de comprimés oubliés

____
(Utiliser la saisie journalière / comptage des comprimés)

Nombre de jours sans médicament

____
(jours)

Poids (kg)

____
(Kg)

Taille (cm) :

____
(CM)

EXAMEN PHYSIQUE

	Bonne Sante	Moderement altere	Mauvais
ETAT GENERAL(Nutritionel)	◯	◯	◯

Please indicate if these examinations were done?

	OUI	NON
VISION NORMALE	○	○
AVEUGLE DES 2 YEUX	○	○
yeux vitreux	○	○
CICATRICES DE BRULURES	○	○
PEAU NORMALE	○	○
PAPULES PRURIGINEUX	○	○
PEAU DE LEOPARD	○	○
PEAU DE LEZARD	○	○
DEMANGEAISONS	○	○
NODULES ONCHOCERCIENS	○	○
CANDIDOSE BUCCALE	○	○
SIGNES MORSURE DE LANGUE	○	○
GANGLIONS CERVICAUX ANORMAUX (>1cm)	○	○

if nodules onchocerciens,nombre de nodules

____

CONSCIENCE

○ alerte ○ Confus ○ Réagit à l'appel ○ Réagit à la douleur

TROUBLE MENTALE ?:

○ NON ○ Peu important (dérange les activités mais encore actif) ○ Important (empêche d'exercer les activités)

Si trouble mental (important ou peu important): lequel? (plusieurs réponses possible)

	Oui	Non	Ne sait pas
difficultés de s'exprimer	○	○	○
désorientation dans le temp	○	○	○
désorientation dans le space	○	○	○
hallucinations	○	○	○
exprime des idées délirantes	○	○	○
ne comprends pas ce qu'on demande	○	○	○
épisodes agressifs	○	○	○
oublie facilement	○	○	○
troubles de comportement	○	○	○

peu d'intérêt ou plaisir dans presque toute activité	○	○	○
autre	○	○	○

Si Autre,specifier

____

Mouvements oculaires

○ Normale ○ altérée ○ NA

Epreuve doigt-nez

○ Normale ○ altérée ○ NA

Marche ?

○ normale ○ base élargie ○ spastique ○ hémiplégique ○ autre

Faiblesse musculaire ?

○ Non ○ Généralisée ○ Localisée

Tonus

○ normal ○ hypotonie ○ hypertonie

EXAMEN NEUROLOGIQUE :

○ NORMAL ○ ABNORMAL

Diagnostique

☐ Epilepsie ☐ Syndrome du hochement de tète ☐ Hochement de tête et autre forme d'épilepsie
☐ Autre

Si autre, spécifier :

○ Convulsions fébriles récurrentes ○ Syncopes ○ Vertiges ○ Anémie sévère ○ retard mental sans épilepsie ○ Hypoglycemie ○ Problème psychiatrique ○ retard mentale important
○ Absences ○ other

Autre

____

PRELEVEMENTS / TESTS

SANG

○ OUI ○ NON ○ NA

Cocher la/les case(s) adaptée(s) au prélèvement :
(Cocher la/les case(s) adaptée(s) au prélèvement :)

☐ papier filtre ☐ tube EDTA

BIOPSIE CUTANEE EXANGUE (SKIN SNIP).

○ OUI ○ NON ○ NA

Date de la biopsie

____
(DD-MM-YYYY)

Heure de le biopsie

____
(HH:MM)

Si OUI, combien de prélèvements ont-ils été effectués ?

☐ Droite ☐ Gauche

Si LECTURE in situ des plaques microtitre, charge microfilarienne

____

Date de lecture

____
(DD-MM-YYYY)

Heure de lecture

____
(HH:MM)

Microfilaire/mg de peau 1

____

Microfilaire/mg de peau 1

____

Biopsie cutanée préservée en tube rempli d'éthanol 80%

○ Oui (droite) ○ Oui(gauche) ○ Non(droite) ○ Non(gauche) ○ Oui (droit et gauche)

Mesure des taux sanguins de médicament anti -épileptique

◯ OUI ◯ NON

Traitement anti -épileptique médicamenteux (décision du médecin)

Changement de la dose

◯ Diminution ◯ Augmentation ◯ Non

Si diminution, specifier raison

◯ Effects secondaires ◯ Autre

Si autre,specifier

Si augmentation, specifier raison

◯ augmentation des crises d'épilepsie ◯ autre

Si, autre, specifier

Nouveau traitement

◯ OUI ◯ NON

Medicament

____

Dose

____

Combien de fois par jours

____

Signature MD/personnel santé

____

Date de la signature

____

Adverse Effects

Signes et symptômes spontanément mentionnés

Le participant a connu un événement indésirable (EI) depuis dernière visite

◯ OUI ◯ NON

EI nombre

____

EI (Événement indésirable) terme

____

Description de l'événement indésirable

EI classement

☐ ----Problèmes gastro-intestinaux---- ☐ Nausées ☐ vomissements ☐ diarrhée ☐ Sécheresse de la bouche ☐ crampes d'estomac ☐ Problèmes gastro-intestinaux - autre, specifier ☐ ----Troubles vasculaires----- ☐ hypotension orthostatique ☐ vascularite cutanée ☐ Troubles vasculaires, autre ,specifier ☐ -----Troubles du système nerveux------ ☐ des étourdissements /Vertiges ☐ maux de tête ☐ encéphalopathie ☐ Somnolence ☐ léthargie ☐ ataxie ☐ nystagmus ☐ somnolence due à l' hyperammoniémie ☐ Troubles du système nerveux - autre, specifier ☐ -----Peau et des tissus sous-cutanés--- ☐ des démangeaisons ☐ urticaire ☐ syndrome de Steven-Johnson ☐ nécrolyse épidermique toxique ☐ Des réactions cutanées ☐ perte de cheveux ☐ Peau et des tissus sous-cutanés, autre ,specifier ☐ ---Troubles musculo-squelettiques et du tissu conjonctif---- ☐ arthralgie ☐ myalgie ☐ Troubles musculo-squelettiques et du tissu conjonctif, autre,specifier ☐ ----Troubles psychiatriques--- ☐ psychose aiguë ☐ Troubles psychiatriques,autre,specifier ☐ ----Trouble de l'oeil----- ☐ conjonctivite ☐ diplopie ☐ ---Trouble de l'oeil, autre précisez--- ☐ --Troubles généraux et administration du site conditions générales---- ☐ fièvre ☐ dème ☐ apathie/fatigue ☐ Troubles généraux et administrationautre,specifier ☐ --Troubles de l'oreille et labyrinthe---- ☐ perte auditive ☐ Troubles de l'oreille et labyrinthe,autre specifier ☐ 1100--Troubles du métabolisme et nutirtional-- ☐ gain de poids ☐ Troubles du métabolisme et nutirtional,autre specifier ☐ --Troubles du système immunitaire--- ☐ réaction allergique cutanée ☐ Troubles du système immunitaire autre,specifier ☐ ---Enquêtes--- ☐ leucopénie ☐ autre specifier ☐ --Sang et troubles du système lymphatique-- ☐ lymphadénite ☐ autre specifier

---

EI autre ,specifier

____

Date de début

____
(DD-MM-YYYY)

L'événement indésirable est-il en cours?

○ OUI ○ NON

Date de fin

____
(DD-MM-YYYY)

Gravité de l' événement indésirable

○ Minimal ○ modérée ○ sévère ○ Vie en danger ○ Mort

Relation avec le traitement

○ Sans aucun doute lié ○ Probablement lié ○ Éventuellement lié ○ Certainement pas lié

Mesures prises

○ aucun ○ Interrompu ○ Discontinué ○ réduit la dose

---

résultat de l'EI (Evenement Indesirable)

○ récupéré ○ récupéré avec séquelles * ○ pas récupéré ○ décès ○ inconnu

---

Evenement indésirable attendu

○ OUI ○ NON

Événement indésirable grave

◯ OUI ◯ NON

Si,Oui ,Événement indésirable grave, décrire

Les effets indésirables supplémentaires

◯ OUI ◯ NON

Initiales du chercheur principal

____

Date d'initiales

____
(DD-MM-YYYY)

SAE Form

Remplissez ce formulaire pour tout événement indésirable qui répond à l'un des critères suivants :

Patient ID

____

Patients initiales

____

Date Participant a rapporté ______________________ (DD-MM-YYYY)

Description de l' EI

Date de début de l'événement indésirable grave ______________________ (DD-MM-YYYY)

Date de la fin de l'événement indésirable grave ______________________ (DD-MM-YYYY)

Etait-ce un effet indésirable inattendu

◯ OUI ◯ NON

Brève description de la nature de l'événement indésirable grave :

CATÉGORIE (résultat) de l'événement indésirable grave :

	Oui	Non	Ne sait pas
décédé	◯	◯	◯
Invalidité/incapacité	◯	◯	◯
danger de mort	◯	◯	◯
Anomalie/malformation congénitale	◯	◯	◯
Hospitalisation	◯	◯	◯

Si Mort, de le mort ______________________ (DD-MM-YYYY)

l'imputabilité de l'événement à l'intervention :

○ non liées ○ possible ○ probable ○ certain

Medicament suspect(s)

	Oui	Non
Ivermectine	○	○
Phenobarbital	○	○
carbamazepine	○	○
valporate de sodium	○	○

Quelles décisions ont été prises avec le traitement à l'étude ?

Résultat

○ récupéré ○ récupéré avec séquelles ○ en cours ○ mort

Si, récupéré, date

____
(DD-MM-YYYY)

Nom en prenon l'enquteur

____

SIGNATURE DE L'ENQUÊTEUR :

____

Date de signature

____
(DD/mm/yyyy)
